# Non-Cancer Causes of Death in Patients With Pancreatic Adenocarcinoma: A Surveillance, Epidemiology, and End Results (SEER)-Based Study

**DOI:** 10.7759/cureus.20289

**Published:** 2021-12-09

**Authors:** Rafique Umer Harvitkar, Harish Peri, Sri Nikhil Zallipalli, Suneet John Joseph, Giri Babu Gattupalli, Khursheed Ansari

**Affiliations:** 1 General Surgery, Queen Alexandra Hospital, Portsmouth, GBR; 2 Department of Surgery, Armed Forces Medical College, Pune, IND; 3 Department of Surgery, Star Hospitals, Hyderabad, IND; 4 Department of Medicine, Armed Forces Medical College, Pune, IND; 5 Surgery, Sri Chandra Sekara Hospital, Hosur, IND; 6 Neurological Surgery, Dr L H Hiranandani Hospital, Mumbai, IND

**Keywords:** seer, mortality, epidemiology, causes of death, pancreatic adenocarcinoma

## Abstract

Background

This study aimed to identify the most common causes of non-cancer mortality in patients with pancreatic adenocarcinoma (PAC) and compare their mortality risk with the general population.

Methodology

This study analyzed PAC patients’ data registered in the Surveillance, Epidemiology, and End Results (SEER) database. We studied the causes of death and investigated their association with age, sex, race, tumor stage at presentation, and treatment modality according to the time interval from diagnosis during which death events occurred. We used the standardized mortality ratio (SMR).

Results

A total of 67,694 PAC patients’ data were analyzed; of these patients, 64,347 (95.06%) died during the follow-up. Most deaths occurred due to cancer (61,685; 95.86% of deaths), while non-cancer mortality represented only 4.14%. The most common causes of non-cancer mortality were heart diseases (SMR = 2.79), cerebrovascular diseases (SMR = 3.11), and septicemia (SMR = 8.2). PAC patients had a higher mortality risk for all studied mortality causes except Alzheimer’s disease (SMR = 0.5) and homicide and legal intervention (SMR = 2.29).

Conclusions

Approximately 96% of PAC patients’ deaths are due to cancer. While the dominant non-cancer causes of death include heart diseases, cerebrovascular diseases, and septicemia, with a higher risk of mortality for most non-cancer causes than the general population.

## Introduction

Pancreatic adenocarcinoma (PAC), the primary cancer of the pancreas, is derived from the exocrine pancreatic cells and accounts for more than 90% of all pancreatic malignancies. According to GLOBOCAN 2018 estimates, it is the eleventh most common cancer and the fourth most frequent cause of cancer mortality worldwide with a five-year overall survival rate of approximately 6% [1-3]. In 2020 alone, there were more than 495,000 new cases and 466,000 deaths of pancreatic cancer worldwide, and the figures are expected to exceed breast cancer as the second most common cause of cancer mortality by 2030 [4,5].

Pancreatic cancer is mainly divided into exocrine cancers (>90% of pancreatic cancers) and endocrine cancers (approximately 7% of pancreatic cancers) [6]. Among exocrine cancers, PAC accounts for 90% of cancers and is the most common. These tumors usually start in the ducts and are called ductal carcinoma. Much less commonly, if the tumor arises from the acini, it is called acinar carcinoma. Other rare exocrine cancers include adenosquamous carcinoma, colloid carcinoma, hepatoid carcinoma, and intraductal papillar mucinous neoplasm (IPMN). Among endocrine cancers, pancreatic neuroendocrine tumors (PanNET) are the most common and constitute 7% of all pancreatic cancers [7,8].

Risk factors for developing pancreatic cancer include tobacco smoking, obesity, diabetes, family history, and chronic pancreatitis [9-11]. The risk in smokers is double or triple that of non-smokers, while heredity increases the risk by tenfold [12,13]. In addition, PAC is more prevalent in developed than in developing countries [14,15].

PAC is a highly devastating disease and has a very poor prognosis with one and five-year survival rates of 24% and 9%, respectively, after diagnosis [16]. The silent nature of the disease and lack of early biomarkers consequently rendering diagnosis in late, unresectable stages are major contributors to the high death rates.

Treatment options include surgical resection, chemotherapy, radiotherapy, immunotherapy, and neoadjuvant therapy. Surgery remains the only treatment choice that targets potential cure. However, most patients are diagnosed late when the tumor is irresectable. Only 10% to 15% of diagnosed PAC patients are candidates for surgery [17]. PAC is considered a systemic disease at the time of diagnosis due to the high rate of micrometastasis at the time of diagnosis. Hence, multimodality therapy is becoming more popular than resection alone [18]. Before surgical resection, neoadjuvant therapy is used to decrease the size of the tumor. Thus, it facilitates resection and improves survival. Moreover, neoadjuvant therapy has been useful for the treatment of early disease when the micrometastases are initially being established [19].

Due to these advances in cancer-directed therapies, the survival rates of pancreatic cancer have marginally increased. However, this has led to a substantial increase in non-cancer deaths in cancer patients [20,21]. Identifying common causes of non-cancer mortality can help improve survival in these patients by controlling these conditions and their related risk factors. These causes are not addressed in a large population-based study, and neither has their risk in PAC patients been identified when compared to the general population.

This retrospective population-based study aims to provide a recent long-term analysis of common non-cancer mortality causes in patients with PAC at different time intervals following diagnosis. In addition, we compared the risk of each cause in these patients with that in the general population.

## Materials and methods

Study design

This retrospective cohort study followed the Strengthening the Reporting of Observational Studies in Epidemiology (STROBE) guidelines [22].

Data sources and study population

We collected data of patients with PAC registered in the Surveillance, Epidemiology, and End Results (SEER) database from 1975 to 2016. Patients’ follow-up data until 2016 or death were analyzed. We considered institutional review board approval non-essential because data registered in the SEER database are anonymous and publicly available. Data extraction was carried out using the SEER*Stat software.

Outcome measured

We studied cancer and non-cancer causes of death in patients with PAC and investigated the possible association of each cause with age, sex, race, tumor stage at diagnosis, and treatment modalities. “Other races” category includes Asian or Pacific Islander and Indian American/Alaska Native. Mortality events were categorized into intervals according to the duration from diagnosis to mortality. These intervals included <1 year, 1-5 years, 5-10 years, and >10 years. For each interval, we reported the overall mortalities and each cause-specific mortality. In addition, we estimated the standardized mortality ratios (SMRs) for each cause of mortality in each interval to compare the risk of death for each cause in PAC patients with that in the general population in the United States (US). Mortality rates for the general US population were obtained from the National Center for Health Statistics from 1969 to 2016 using the SEER*Stat.

Statistical analysis

We estimated SMRs with 95% confidence intervals (CIs) and analyzed data using the SEER*Stat software (version 8.3.9). High mortality risk was considered when the observed mortality events for a specific cause in PAC patients are significantly higher than the expected mortality events for the same cause in the general population (p < 0.05). Two-sided statistical tests were performed in this study.

## Results

Baseline characters

We included 67,694 PAC patients in this study. Most patients were aged ≥50 years at diagnosis (63,072; 93.17%), males (34,885; 51.53%), White (54,145; 79.98%), and with distant tumor stage (40,814; 60.29%). Most patients received chemotherapy (37,989; 56.12%), but a minority had radiotherapy (12,521; 18.5%) or surgery (11,250; 16.62%).

During the follow-up period, 64,347 (95.06%) patients died. Most deaths occurred during the first year after diagnosis (48,723; 75.72% of deaths). Events of death decreased with time after diagnosis, with 15,057 deaths occurring during the 1-5-year interval (23.4% of deaths), 489 deaths during the 5-10-year intervals (0.76% of deaths), and 78 deaths after >10 years (0.12% of deaths) following PAC diagnosis. Details of the characteristics of the total study population and patients who died in each time interval are shown in Table 1.

**Table 1:** Baseline characteristics of pancreatic adenocarcinoma patients. ^a^: Asian or Pacific Islander and Indian American/Alaska Native patients

Characteristic	<1 year	1–5 years	5–10 years	>10 years	All deaths	Total number of patients
	No. of Deaths (%)	No. of Deaths (%)	No. of Deaths (%)	No. of Deaths (%)	No. of Deaths (%)	
Overall	48,723	15,057	489	78	64,347 (100%)	67,694
Age						
<50 years	2,967	1,297	44	4	4,312 (100%)	4,622
≥50 years	45,756	13,760	445	74	60,035 (100%)	63,072
Sex						
Male	25,337	7,624	228	39	33,228 (100%)	34,885
Female	23,386	7,433	261	39	31,119 (100%)	32,809
Race						
White	38,744	12,269	422	60	51,495 (100%)	54,145
Black	6,503	1,727	44	12	8,286 (100%)	8,661
Other^a^	3,476	1,061	23	6	4,566 (100%)	4,888
Stage						
Localized	2,915	1,376	109	16	4,416 (100%)	4,965
Regional	11,982	7,808	317	49	20,156 (100%)	21,915
Distant	33,826	5,873	63	13	39,775 (100%)	40,814
Surgery						
Yes	3,553	4,608	373	61	8,595 (100%)	11,250
Chemotherapy						
Yes	23,135	11,998	324	45	35,502 (100%)	37,989
Radiotherapy						
Yes	6,027	5,214	216	37	11,494 (100%)	12,521

Causes of death

Most deaths in the study population were attributed to cancer (61,685; 95.86%), mainly pancreatic cancer (58,670; 91.18%). Non-cancer causes of death represented 4.14% of the total deaths (2,662 deaths). Heart diseases represented the most dominant non-cancer cause of mortality (843; 1.31%) (SMR = 2.79; 95% CI = 2.6-2.98), followed by cerebrovascular diseases (212; 0.33%) (SMR = 3.11; 95% CI = 2.71-3.56), and septicemia (140; 0.22%) (SMR = 8.2; 95% CI = 6.9-9.68). The risk of mortality due to Alzheimer’s disease was lower in PAC patients than in the general population (SMR = 0.5; 95% CI = 0.3-0.78), while the risk was comparable in both populations for homicide and legal intervention (SMR = 2.29; 95% CI = 0.47-6.68). The mortality risk was higher in PAC patients than in the general population for other studied causes of mortality. Table 2 shows the observed death events with the SMRs for each cause of mortality during the follow-up period and each time interval after diagnosis.

Causes of death in the first year after diagnosis

A total of 48,723 patients died in the first year after PAC diagnosis. Of these patients, 44,388 (91.1%) died due to pancreatic cancers, 2,427 (4.98%) died due to other cancers, and 1,908 (3.92%) died due to non-cancer causes, with a higher mortality risk than the general population (Table 2). The most common non-cancer cause of mortality was heart diseases (620; 1.27%) (SMR = 3.17; 95% CI = 2.93-3.43), followed by cerebrovascular diseases (163; 0.33%) (SMR = 3.70; 95% CI = 3.15-4.31), and septicemia (109; 0.22%) (SMR = 9.90; 95% CI = 8.13-11.95). The risk of death due to Alzheimer’s disease was lower in PAC patients than in the general population (SMR = 0.29; 95% CI = 0.12-0.6).

**Table 2:** Standardized mortality ratios for each cause of death categorized by the timing of deaths after pancreatic adenocarcinoma diagnosis. SMR: standardized mortality ratio; CI: confidence interval; HIV: human immunodeficiency virus; ICD: International Classification of Diseases

Cause of death	<1 year		1–5 years		5–10 years		>10 years		Total	
	Observed	SMR (95% CI)	Observed	SMR (95% CI)	Observed	SMR (95% CI)	Observed	SMR (95% CI)	Observed	SMR (95% CI)
All causes of death	48,723	66.45 (65.86-67.05)*	15,057	43.31 (42.62-44.01)*	489	10.00 (9.13-10.92)*	78	8.59 (6.79-10.73)*	64,347	56.50 (56.07-56.94)*
Pancreatic cancers	44,388	3,799.65 (3,764.38-3,835.16)*	13,951	2,481.09 (2,440.09-2,522.61)*	305	451.42 (402.17-505.03)*	26	229.69 (150.04-336.55)*	58,670	3,242.53 (3,216.34-3,268.87)*
Other cancer deaths	2,427	83.54 (80.25-86.93)*	517	24.69 (22.61-26.91)*	53	5.94 (4.45-7.78)*	18	9.14 (5.41-14.44)*	3,015	49.52 (47.77-51.32)*
All non-cancer causes of death	1,908	46.39 (44.33-48.52)*	589	10.89 (10.03-11.81)*	131	5.08 (4.25-6.03)*	34	6.15 (4.26-8.59)*	2,662	21.04 (20.25-21.85)*
In situ, benign or unknown behavior neoplasm	52	11.32 (8.45-14.84)*	21	9.50 (5.88-14.52)*	1	3.11 (0.08-17.34)	0	0 (0-61.12)	74	10.30 (8.08-12.92)*
Tuberculosis	0	0 (0-18.47)	0	0 (0-41.1)	0	0 (0-366.36)	0	0 (0-2,328.92)	0	0 (0-12.25)
Syphilis	0	0 (0-315.32)	0	0 (0-707.45)	0	0 (0-5,882.37)	0	0 (0-35,934.19)	0	0 (0-209.08)
Septicemia	109	9.90 (8.13-11.95)*	27	5.18 (3.41-7.53)*	4	5.59 (1.52-14.32)*	0	0 (0-28.56)	140	8.20 (6.9-9.68)*
Other infectious and parasitic diseases including HIV	44	6.78 (4.92-9.1)*	11	3.59 (1.79-6.42)*	2	5.35 (0.65-19.34)	3	46.74 (9.64-136.6)*	60	6.00 (4.58-7.73)*
Diabetes mellitus	61	2.60 (1.99-3.34)*	20	1.82 (1.11-2.81)*	10	7.30 (3.5-13.42)*	2	8.54 (1.03-30.87)*	93	2.58 (2.08-3.16)*
Alzheimer’s (ICD-9 and 10 only)	7	0.29 (0.12-0.6)*	4	0.35 (0.09-0.89)*	6	2.66 (0.98-5.79)	2	3.9 (0.47-14.07)	19	0.50 (0.3-0.78)*
Heart diseases	620	3.17 (2.93-3.43)*	183	2.00 (1.72-2.32)*	35	2.65 (1.84-3.68)*	5	2.05 (0.66-4.78)	843	2.79 (2.6-2.98)*
Hypertension without heart disease	30	3.73 (2.51-5.32)*	3	0.79 (0.16-2.3)	1	1.63 (0.04-9.1)	3	24.02 (4.95-70.19)*	37	2.94 (2.07-4.05)*
Cerebrovascular diseases	163	3.70 (3.15-4.31)*	38	1.86 (1.32-2.55)*	9	2.98 (1.36-5.66)*	2	3.47 (0.42-12.54)	212	3.11 (2.71-3.56)*
Atherosclerosis	13	4.79 (2.55-8.19)*	3	2.45 (0.5-7.15)	0	0 (0-19.71)	0	0 (0-111.89)	16	3.84 (2.2-6.24)*
Aortic aneurysm and dissection	8	2.03 (0.88-4)	4	2.19 (0.6-5.6)	0	0 (0-16.22)	1	28.51 (0.72-158.88)	13	2.16 (1.15-3.69)*
Other diseases of arteries, arterioles, capillaries	11	3.72 (1.86-6.65)*	2	1.45 (0.18-5.22)	1	5 (0.13-27.85)	0	0 (0-101.22)	14	3.06 (1.67-5.13)*
Pneumonia and influenza	37	2.14 (1.51-2.95)*	19	2.34 (1.41-3.65)*	6	4.75 (1.74-10.34)*	2	8.50 (1.03-30.72)*	64	2.38 (1.83-3.03)*
Chronic obstructive pulmonary disease and allied conditions	73	1.58 (1.24-1.99)*	19	0.85 (0.51-1.32)	13	4.19 (2.23-7.16)*	1	1.8 (0.05-10.03)	106	1.47 (1.2-1.77)*
Stomach and duodenal ulcers	14	13.63 (7.45-22.88)*	3	6.32 (1.3-18.46)*	1	16.26 (0.41-90.6)	0	0 (0-347.13)	18	11.44 (6.78-18.08)*
Chronic liver disease and cirrhosis	32	4.30 (2.94-6.07)*	15	4.26 (2.39-7.03)*	8	24.73 (10.67-48.72)*	3	69.59 (14.35-203.37)*	58	5.12 (3.89-6.62)*
Nephritis, nephrotic syndrome and nephrosis	34	2.30 (1.59-3.22)*	11	1.57 (0.78-2.8)	4	3.85 (1.05-9.86)*	1	5.21 (0.13-29.03)	50	2.17 (1.61-2.86)*
Complications of pregnancy, childbirth, puerperium	8	869.71 (375.48-1,713.68)*	0	0 (0-912.31)	0	0 (0-22,917.93)	0	0 (0-283,758.45)	8	596.31 (257.44-1,174.97)*
Congenital anomalies	4	6.08 (1.66-15.57)*	2	6.51 (0.79-23.52)	0	0 (0-118.17)	0	0 (0-769.8)	6	5.99 (2.2-13.05)*
Certain conditions originating in perinatal period	0	0 (0-1,466.42)	0	0 (0-3,176.38)	0	0 (0-33,828.67)	0	0 (0-262,775.03)	0	0 (0-970.76)
Symptoms, signs and ill-defined conditions	52	6.50 (4.86-8.53)*	13	3.41 (1.81-5.82)*	2	2.98 (0.36-10.77)	1	7.2 (0.18-40.11)	68	5.39 (4.18-6.83)*
Accidents and adverse effects	66	3.41 (2.64-4.34)*	25	2.72 (1.76-4.01)*	3	2.47 (0.51-7.22)	2	8.72 (1.06-31.51)*	96	3.20 (2.59-3.91)*
Suicide and self-inflicted injury	53	11.57 (8.66-15.13)*	14	6.50 (3.55-10.9)*	0	0 (0-18.39)	0	0 (0-141.21)	67	9.62 (7.46-12.22)*
Homicide and legal intervention	3	3.38 (0.7-9.88)	0	0 (0-9.47)	0	0 (0-122.36)	0	0 (0-857.34)	3	2.29 (0.47-6.68)
Other cause of death	414	3.97 (3.6-4.38)*	152	3.02 (2.56-3.54)*	25	3.07 (1.99-4.53)*	6	3.52 (1.29-7.67)*	597	3.63 (3.35-3.94)*

Regarding specific demographic and pathological subgroups, patients aged <50 years at diagnosis showed more deaths due to infectious and parasitic diseases than cerebrovascular diseases. Black patients showed more deaths due to septicemia than cerebrovascular diseases, while Other races showed more deaths due to pneumonia and influenza than septicemia. Patients presenting with localized disease showed deaths due to chronic obstructive pulmonary disease (COPD) as well as accidents and adverse events equal to that due to septicemia. Patients treated with radiotherapy showed more deaths due to septicemia than cerebrovascular diseases. Other subgroups showed mortality trends similar to that of the overall population.

Causes of death at 1-5 years after diagnosis

During the period of one to five years after PAC diagnosis, 15,057 patients died. Of these patients, 13,951 (92.65%) died due to pancreatic cancer, 517 (3.43%) died due to other cancers, and 589 (3.91%) died due to non-cancer cause; and the risk of mortality was significantly high (Table 2). The most common non-cancer mortality causes were heart diseases (183 deaths; 1.21%) (SMR = 2.00; 95% CI = 1.72-2.32), cerebrovascular diseases (38 deaths; 0.25%) (SMR = 1.86; 95% CI = 1.32-2.55), and septicemia (27 deaths; 0.18%) (SMR = 5.18; 95% CI = 3.41-7.53). The risk of Alzheimer’s disease mortality was significantly low (SMR = 0.35; 95% CI = 0.09-0.89).

Regarding specific patients’ subgroups, females showed more deaths due to pneumonia and influenza than due to septicemia; White patients showed more deaths due to accidents and adverse events than from septicemia; Black patients showed deaths due to infectious and parasitic diseases equal to that due to cerebrovascular diseases and septicemia; Other races showed more deaths due to diabetes than cerebrovascular diseases and more deaths due to pneumonia and influenza than septicemia; and patients with distant tumor stage showed more deaths due to diabetes, pneumonia and influenza, COPD, nephritis and nephrosis, suicide, accidents and adverse events, and due to symptoms, signs, and ill-defined conditions than deaths due to septicemia. In patients with localized disease, diabetes was the second leading cause of non-cancer mortality after heart diseases, followed by chronic liver diseases, pneumonia and influenza, COPD, accidents and adverse events, and septicemia. Patients who underwent chemotherapy showed deaths due to Alzheimer’s disease, COPD, and chronic liver disease similar to that due to septicemia, while patients who underwent radiotherapy showed more deaths due to septicemia than due to cerebrovascular diseases. Other patients’ subgroups showed similar mortality trends as the general population.

Causes of death at 5-10 years following diagnosis

A total of 489 PAC patients died at 5-10 years. Of these patients, 305 (62.37%) died from pancreatic cancer, 53 (10.84%) died due to other cancers, and 131 (26.79%) died due to non-cancer causes, with a higher risk of mortality than that in the US general population (Table 2). The leading causes of non-cancer mortality included heart diseases (35 deaths; 7.16%) (SMR = 2.65; 95% CI = 1.84-3.68), COPD (13 deaths; 2.66%) (SMR = 4.19; 95% CI = 2.23-7.16), and diabetes (10 deaths; 2.04%) (SMR = 7.30; 95% CI = 4.5-13.42).

White patients and patients with localized disease at diagnosis showed deaths due to chronic liver diseases equal to those due to diabetes, while Blacks showed more deaths due to infectious and parasitic diseases (including HIV) more than any other non-cancer causes. In Other races, patients with regional disease and those treated with chemotherapy, heart diseases, and cerebrovascular diseases were the leading causes of non-cancer mortality. Diabetes was the most common cause of non-cancer death in patients who presented with distant tumor stage. Patients treated with surgery had more deaths due to cerebrovascular diseases than diabetes. Patients who had chemotherapy showed more deaths due to septicemia, Alzheimer’s disease, and chronic liver diseases than diabetes. Patients treated with radiation showed more deaths due to septicemia, Alzheimer’s disease, nephritis and nephrosis, and chronic liver diseases than diabetes. Other patients’ subgroups showed a similar trend to that of the general population.

Causes of death after more than 10 years following diagnosis

Only 78 deaths occurred after more than 10 years of PAC diagnosis. Of these, 26 (33.3%) occurred due to pancreatic cancers, 18 (23.08%) due to other cancers, and 34 (43.59%) due to non-cancer causes; and the mortality risk was high (Table 2). The most common non-cancer causes of death were heart diseases (five deaths; 6.41%) (SMR = 2.05; 95% CI = 0.66-4.78), infectious and parasitic diseases (three deaths; 3.85%) (SMR = 46.74; 95% CI = 9.64-136.6), hypertension (three deaths; 3.85%) (SMR = 24.02; 95% CI = 4.95-70.19), and chronic liver diseases (three deaths; 3.85%) (SMR = 69.59; 95% CI = 14.35-203.37).

Heart diseases and infectious and parasitic diseases were the leading causes of non-cancer mortality in males. At the same time, it was hypertension followed by heart diseases and pneumonia and influenza in females. In patients with localized disease and those treated with chemotherapy or radiotherapy, chronic liver disease was the most common cause of non-cancer deaths. Other subgroups showed similar rates as the general population or had a low number of deaths to conclude results.

## Discussion

The present study analyzed data of 67,694 PAC patients showed that about 96% of PAC patients died due to cancer-related causes, while non-cancer causes represented only about 4% of deaths. The most common non-cancer causes of death were heart diseases, cerebrovascular diseases, and septicemia. The risk of mortality due to cancer and non-cancer causes was higher in PAC patients than in the general population, except for homicide and legal intervention, which was similar in both populations, and for Alzheimer’s disease, which was lower in PAC patients.

Previous studies reported that PAC is typically diagnosed in old age, mostly in the seventh or the eighth decade, and about 90% of cases are diagnosed after the age of 55 [23,24]. Our results coincide with these findings, with more than 93% of patients diagnosed after 50 years of age.

The most critical prognostic factor in pancreatic cancer is the disease stage at presentation [7]. But unfortunately, a minority of patients present with surgically resectable disease [4,7]. Patients undergoing surgical resection have a five-year survival of 27% [25]. Our study revealed a similar result, with 27.5% of patients who underwent surgery surviving for more than five years after PAC diagnosis. On the other hand, patients presenting with a metastatic or locally invasive irresectable disease have a median survival not exceeding 11 months [25]. Surgical resection remains the only potentially curative choice for pancreatic cancers, with the addition of chemotherapy as an adjuvant therapy to improve survival [1,4].

Only about 17% of included patients in our study underwent cancer-directed surgery. This low percentage is primarily due to the late diagnosis and the aggressive nature of the disease [7]. PAC is usually diagnosed when the tumor is locally invasive or metastatic, where surgery has no curative role [26]. In our study, most patients (about 60%) had a distant tumor stage at presentation. In addition, most included patients (about 72%) died during the first year after diagnosis. About 94% died during the first five years after diagnosis, reflecting the aggressive nature and the poor prognosis of the disease. Hence, our results revealed an abysmal survival rate in PAC patients, with a five-year survival rate of less than 6%.

Death due to diabetes is more likely to occur in cancer patients than in the general population [27]. Moreover, pancreatic cancer is among the most likely cancers to increase diabetes mortality. In our study, diabetes mortality was significantly higher in PAC patients than in the general population throughout all time intervals after PAC diagnosis.

A previous meta-analysis showed that suicide mortality is higher in cancer patients than in the general population [28]. Our study showed similar results for the first five years after diagnosis but showed no events of suicide death in PAC patients after more than five years of PAC diagnosis.

Heart diseases remained the most typical cause of non-cancer mortality across all periods after PAC diagnosis. Moreover, an earlier study named heart diseases as the first cause of non-cancer mortality in many types of cancer, including pancreatic cancer [29]. The same study reported that pancreatic cancer is among the most likely cancer associated with cancer mortality [29].

Strengths and limitations

This study includes a large sample of patients with highly reliable data based on the SEER registry. In addition, we analyzed cancer and non-cancer causes of mortality in the studied population. However, some patient subpopulations had a small number of patients, limiting the generalizability of the results to their corresponding subgroups. In addition, the study’s retrospective design and the absence of personal and environmental data that may affect PAC outcomes are other limitations.

## Conclusions

Most patients with PAC die due to cancer. The dominant causes for non-cancer mortality include heart diseases, cerebrovascular diseases, and septicemia. The risk of death due to cancer and several non-cancer causes is higher in PAC patients than in the general population.
